# Editorial: Targeting mitochondrial dysfunction for the diagnosis and treatment of Alzheimer’s and cognitive-related diseases

**DOI:** 10.3389/fphar.2024.1455646

**Published:** 2024-07-15

**Authors:** Manos C. Vlasiou, Lefteris C. Zacharia, Eleni Douni, Christos Papaneophytou

**Affiliations:** ^1^ School of Veterinary Medicine, University of Nicosia, Nicosia, Cyprus; ^2^ Department of Health Sciences, School of Life and Health Sciences, University of Nicosia, Nicosia, Cyprus; ^3^ Bioactive Molecules Research Center, School of Life and Health Sciences, University of Nicosia, Nicosia, Cyprus; ^4^ Institute for Bioinnovation, Biomedical Sciences Research Center “Alexander Fleming”, Vari, Greece; ^5^ Laboratory of Genetics, Department of Biotechnology, Agricultural University of Athens, Athens, Greece; ^6^ Department of Life Sciences, School of Life and Health Sciences, University of Nicosia, Nicosia, Cyprus

**Keywords:** Alzheimer’s disease, dementia, neurocognitive diseases, mitochondrial dysfunction, phosphorylation

With a continuously growing aging population, neurocognitive diseases are becoming a global concern, affecting not only patients but also their caregivers, and significantly pressuring healthcare systems and the economy (Feigin et al., 2020). Despite extensive research efforts and many clinical trials, Alzheimer’s disease—the most prevalent form of dementia—still lacks effective drugs to slow its progression or treat the condition (Moutinho, 2022). Early diagnosis of Alzheimer’s disease and other neurocognitive disorders, even before the initial signs of dementia, is believed to be crucial in preventing disease progression (Juganavar et al., 2023). Additionally, many drugs in clinical trials fail to demonstrate efficacy because they are tested on patients with advanced forms of dementia. Therefore, early diagnosis of neurocognitive disorders is as crucial as developing effective treatments (Bishnoi, 2023).

Identifying and targeting complex pathological mechanisms for treatment or diagnosis is vital in addressing neurocognitive diseases (Khan and Al-Jahdali, 2023). Many hypotheses for the pathogenesis of cognitive disorders, including Alzheimer’s disease, have been proposed, with various dysfunctional cellular processes implicated. Healthy mitochondria support neuronal activity by providing energy, protecting neurons from oxidative damage, and regulating apoptosis. Recent evidence implicates mitochondrial dysfunction as a common pathological mechanism alongside well-established pathological processes, such as amyloid-beta (Aβ) aggregate formation, neurofibrillary tangles caused by tau aggregates, impaired synaptic transmission, oxidative stress, neurodegeneration, and neuroinflammation (Clemente-Suárez et al., 2023). Exploring the mitochondrial processes altered during the pathogenesis of neurocognitive diseases, such as structural and functional changes, may offer promising therapeutic targets for diagnosis and treatment (Yang et al., 2021).

This Research Topic aimed to gather publications studying mitochondrial dysfunction in Alzheimer’s disease and other cognitive decline disorders such as Huntington’s, Lewy Body, and Parkinson’s disease. The focus was on identifying new targets, therapeutics, and potential novel biomarkers for cognitive diseases. The contributions in this Research Topic provide new insights into mitochondrial dysfunction in neurocognitive disorders and significantly enhance the field’s knowledge.

Accordingly, in their study, Zhao et al. explored the protective roles of malvidin in sepsis-associated encephalopathy (SAE) and its underlying mechanisms. They developed SAE mouse models and treated them with malvidin, observing restored neurobehavioral function, reduced serum S100 calcium binding protein β (S100β) and neuron specific enolase (NSE) levels, maintained cerebral structure, improved blood-brain barrier integrity, and decreased Evans blue leakage, protecting the mice from brain injury. Malvidin prevented mitochondrial dysfunction by enhancing JC-1 aggregates and ATP levels, and reducing reactive oxygen species (ROS) accumulation through decreased lipid peroxidation and increased antioxidant enzymes. It restored uncoupling protein 2 (UCP2) protein levels, which had decreased after LPS stimulation. Inhibiting UCP2 disrupted mitochondrial potential and decreased ATP levels, indicating UCP2 as a key target. Dorsomorphin block assays confirmed that malvidin upregulated UCP2 via AMPK phosphorylation. Additionally, malvidin alleviated SAE by inhibiting ROS-dependent NLRP3 inflammasome activation, reducing pro-inflammatory cytokine secretion, and decreasing mitochondrial apoptosis, with lower Bax, cytochrome C, caspase-3, and higher Bcl-2 levels. In conclusion, malvidin acts on the AMPK-α/UCP2 axis to mitigate mitochondrial dysfunction and ROS buildup, inhibiting NLRP3 inflammasome activation and apoptosis, thus protecting against SAE.

Guo et al. conducted a bibliometric analysis of global research on mitochondrial dysfunction in Alzheimer’s disease from 2013 to 2022, mapping trends and hotspots. This study summarized research hotspots and trends in this field over the past decade. The number of publications on mitochondrial dysfunction and AD increased until 2021, with a slight decrease in 2022. The United States leads in publications, H-index, and international cooperation in this research. Texas Tech University has the most publications, while the Journal of Alzheimer’s Disease and Oxidative Medicine and Cellular Longevity are prominent journals in this field. Autophagy, mitochondrial autophagy, and neuroinflammation are emerging research hotspots. This study highlighted the current research trajectory concerning mitochondrial dysfunction in AD.

Hyperphosphorylation of tau, leading to neurofibrillary tangles, is a key event in AD pathogenesis. Microtubule-affinity regulating kinase 4 (MARK4) phosphorylates tau and is implicated in AD pathology (Basheer et al., 2023). Inhibiting MARK4 is an attractive therapeutic strategy. In their study, Adnan et al. examined mechanistic insights into MARK4 inhibition by galantamine toward therapeutic targeting of Alzheimer’s disease. Molecular docking, molecular dynamic studies, fluorescence binding, and isothermal titration calorimetry demonstrated that galantamine (GLT), an acetylcholinesterase inhibitor, binds to MARK4 with strong affinity and inhibits its kinase activity. This suggests GLT is a potential MARK4 inhibitor and a therapeutic target for AD.

The link between sleep deprivation and cognitive decline is well-documented, yet the precise mechanisms and potential biomarkers involved remain incompletely understood. Liu et al. investigated shared biological mechanisms and common biomarker ATPAF1 in sleep deprivation and mild cognitive impairment using integrated bioinformatics analysis. The study identified differentially expressed mitochondrial-related genes in both conditions, with ATPAF1 emerging as a common biomarker. Functional analysis revealed ATPAF1 involvement in metabolic pathways and immune cell infiltration changes. This study provided insights into mitochondrial dysfunction as a common pathogenesis of sleep loss and cognitive impairment and identified ATPAF1 as a potential biomarker and therapeutic target.

In conclusion, this Research Topic emphasizes that targeting mitochondrial dysfunction holds significant potential for diagnosing and treating Alzheimer’s and other cognitive-related diseases. As research continues to elucidate the complex role of mitochondria in these disorders, it paves the way for novel therapeutic strategies and diagnostic tools. Early diagnosis and intervention are critical for effective management, and a deeper understanding of mitochondrial processes offers a pathway to innovative treatments. This Research Topic successfully highlights the importance of exploring mitochondrial dysfunction and encourages further research to identify new targets, therapies, and biomarkers that could revolutionize the approach to neurocognitive diseases. These advancements could complement other emerging therapies, enhancing symptom relief and slowing disease progression. Importantly, a contribution from Adnan et al. within this Research Topic underscores the potential of “multi-target drug ligands,” which can act on multiple targets, showing better efficacy in complex neurodegenerative diseases. For example, Galantamine, traditionally known as an acetylcholinesterase inhibitor for symptomatic treatment of AD, was shown by Adnan et al. to also inhibit MARK4, potentially preventing tau hyperphosphorylation, a key mechanism in AD pathogenesis.
